# Phlorizin Ameliorates Amyloid-β Toxicity and Enhances Fatty Acid β-Oxidation in *Caenorhabditis elegans* via NHR-49-Dependent Pathway

**DOI:** 10.3390/ijms26199303

**Published:** 2025-09-23

**Authors:** Xuya Zhang, Yan Fu, Xue Li, Yali Zhang, Lingling Li, Tianxing Yi, Hong Jiang, Yi Lu

**Affiliations:** School of Traditional Chinese Medicine, Beijing University of Chinese Medicine, Beijing 100105, China; 20220931137@bucm.edu.cn (X.Z.); f1500292657@163.com (Y.F.); crzay9@126.com (X.L.); zhangyalichn@163.com (Y.Z.); linglingl2023@163.com (L.L.); ytx18515831039@163.com (T.Y.); 18282643481@163.com (H.J.)

**Keywords:** Phlorizin, *Caenorhabditis elegans*, Alzheimer’s disease, fatty acid β-oxidation, lipid metabolism

## Abstract

Phlorizin (PHZ) is a glucoside of phloretin, belonging to the dihydrochalcone class within flavonoids; It is one of the active ingredients of the plant Cynomorium, and it has been shown that PHZ can regulate lipid metabolism disorders as well as having anti-aging properties. However, no studies have investigated whether PHZ ameliorates Aβ-induced toxicity in Alzheimer’s disease (AD) by regulating fatty acid β-oxidation. This study aims to investigate the effects of PHZ on the regulation of fatty acid β-oxidation and resistance to Aβ-associated toxicity on the AD *Caenorhabditis elegans* and the mechanisms of action. Wild-type N2 and AD model CL4176 *C. elegans* were used; lifespan, heat stress resistance, chronic paraquat stress, reactive oxygen species (ROS), behavioral performance, and lipofuscin accumulation assays were examined to evaluate the anti-aging effects; and non-esterified fatty acid (NEFA), triglyceride (TG) and lipidomic contents were quantified after PHZ treatment. The detection of genes related to fatty acid β-oxidation pathways was performed using qRT-PCR. *nhr-49* knockout mutant RB1716; and GFP-binding mutants PMD150 WBM170 were used to observe the effect of PHZ on NHR-49 pathways, and molecular docking studies were performed by combining PHZ with NHR-49 proteins. Results showed that PHZ improved worms’ survival and delayed senescence, as demonstrated by enhanced performance in lifespan, heat stress, ROS, and paraquat assays and chronic paraquat assays; PHZ also reduced lipid accumulation in worms, affected the unsaturated fatty acid pathway, and significantly increased the expression of fatty acid metabolism-related genes *nhr-49*, *acs-2*, and *cpt-5*, and can be tightly coupled to NHR-49 targets. PHZ may play an anti-Aβ toxicity role by regulating lipid metabolism disorders through the NHR-49—related pathway and anti-aging in AD worms.

## 1. Introduction

Alzheimer’s disease (AD) is the most common age-related neurodegenerative disorder, characterized by the accumulation of beta-amyloid (Aβ) plaques and neurofibrillary tangles [1]. Aβ formation results from the amyloidogenic cleavage of human amyloid precursor protein (APP). Aβ is toxic to neurons through multiple mechanisms. It can cause pore formation resulting in the leakage of ions, disruption of cellular calcium balance, and loss of membrane potential. It can promote apoptosis, cause synaptic loss, and disrupt the cytoskeleton [2].

These pathological features are accompanied by significant alterations in lipid metabolism and behavioral impairments in patients [3]. Studies have demonstrated that lipid metabolism and its associated pathways play a critical role in the etiology of late-onset AD [4,5,6]. Aging is the largest known risk factor for AD aside from genetic predisposition, as it triggers extensive changes at the cellular and systemic levels [7]. While the mechanisms linking aging to AD are complex, clinical evidence suggests that compounds capable of slowing aging and extending lifespan may be effective in combating Alzheimer’s disease [8].

Phlorizin is a glucoside of phloretin and belongs to the dihydrochalcone class within flavonoids [9]. PHZ displays multifaceted biological activities, notably anti-aging properties. Previous studies have established the efficacy of glycosides in modulating Alzheimer’s disease (AD) pathogenesis [10]. PHZ belongs to the dihydrochalcone subclass of flavonoids [11] and is widely recognized for its regulatory roles in glucose and lipid metabolism, with demonstrated therapeutic potential against diabetes [12]. Additionally, PHZ has been shown to improve brain function [13]. PHZ is a competitive inhibitor of sodium-glucose co-transporters SGLT1 and SGLT2 [14]; it reduces renal glucose transport and lowers blood glucose. These findings align with earlier reports highlighting PHZ’s pharmacological efficacy in ameliorating lipid metabolism dysregulation [15]. Current research indicates that PHZ exhibits antioxidant and anti-inflammatory effects, enhances cholinergic signaling activity in the hippocampus and cerebral cortex, and provides neuroprotection against LPS-induced neuroinflammation and memory impairment. PHZ can also alleviate metabolic disorders and gene expression imbalances induced by high glucose, and inhibit oxidative stress and lipid accumulation [16,17,18]. However, limited research has explored the relevance of PHZ in mitigating Aβ-induced toxicity through lipid metabolism modulation.

The primary objective of this study was to investigate the anti-aging pharmacological efficacy of PHZ and its potential role in counteracting amyloid-β (Aβ) toxicity, with a specific focus on elucidating its regulatory effects on fatty acid β-oxidation using the *C. elegans* model. Our findings demonstrate that PHZ mitigates Aβ-induced neurotoxicity by modulating fatty acid β-oxidation, thereby conferring neuroprotective effects. To further unravel the mechanistic basis of PHZ action, we employed mutant *C. elegans* strains targeting signaling pathways associated with fatty acid β-oxidation dysregulation. Collectively, our results suggest that PHZ holds promise as a therapeutic candidate for AD.

## 2. Result

### 2.1. PHZ Concentration-Dependently Increased C. elegans’ Resistance

**Figure 1:**
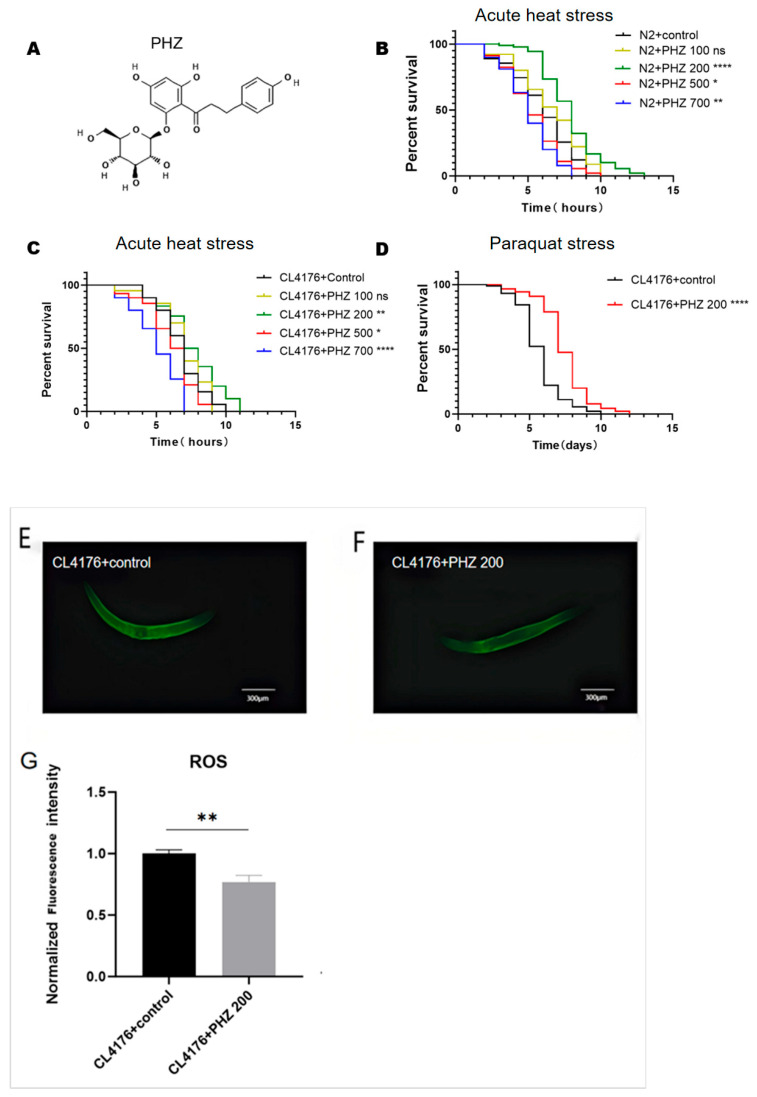
A quantity of 200 μM PHZ significantly enhances the resistance of *C. elegans*. (**A**) Structural formula of PHZ. (**B**) Survival curve of N2 acute heat stress after treatment with different concentrations of PHZ. (**C**) Survival curve of CL4176 after treatment with different concentrations of PHZ. (**D**) Survival curve of CL4176 treated with 200 μM PHZ for chronic paraquat stress; CL4176 treated with PHZ for 24 h followed by heat stress at 35 °C for 30 min. (**E**,**F**) Representative fluorescent images (10×) depicting of the control group and the PHZ group. (**G**) ImageJ (Pro Plus 6.0) quantitative analysis; **** (*p* < 0.0001), ** (*p* < 0.01) and * (*p* < 0.05).

The resistance of *C. elegans* to external stimuli decreases dramatically with aging [19], as evidenced by the shortened lifespan under acute heat stress conditions. Therefore, heat stress resistance testing is a rapid screening method for drugs and their effective concentration [20]. In this experiment we used 35 °C as an induction of heat stress for the experiment. We found that among the five treatment groups, the most significant prolongation of the worm lifespan was in the 200 μM PHZ group (Figure 1B, Table 1), (33.03%, *p* < 0.0001). We then observed the resistance of the Aβ1-42 transgenic nematode CL4176. Interestingly, under heat stress conditions, 200 μM PHZ showed a similar effect (12.26%, *p* < 0.01) on CL4176 to improve resistance in N2 (Figure 1C, Table 1). Based on these results, 200 μM PHZ was used in the following stages.

Oxidative stress is also a major cause of aging, in order to test the antioxidant effect of PHZ under environmental stress, we used 4 mM paraquat to induce chronic oxidative stress, and chronic paraquat oxidative stress experiments showed that 200 μM PHZ increased the *C. elegans*’s resistance (*p* < 0.0001) to oxidative stress (Figure 1D, Table 2).

After 24 h treatment with 200 μM PHZ, CL4176 worms subjected to 35 °C acute heat stress for 30 min exhibited significantly lower mean fluorescence intensity compared to the control group (Figure 1E–G), indicating that 200 μM PHZ effectively eliminates excessive reactive oxygen species (ROS) in *C. elegans* (*p* < 0.01).

### 2.2. PHZ Extends the Lifespan of Wild-Type N2 and Transgenic C. elegans CL4176

**Figure 2:**
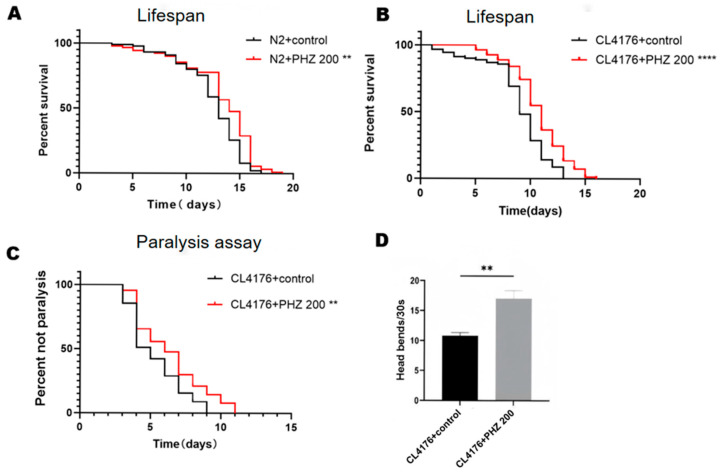
PHZ prolongs the lifespan of *C. elegans* and protects against Aβ- mediated paralysis and behavioural impairments. (**A**) PHZ prolongs the survival of the wild-type N2 worms. (**B**) PHZ prolongs the survival of CL4176. (**C**) PHZ protects against Aβ-induced paralysis. (**D**) PHZ increases the rate of head-bending movements of CL4176; ** (*p* < 0.01) **** (*p* < 0.0001).

The free radical theory of aging perceives that aging results from free radical reactions [21]. and also several nutritional antioxidants function as free radical scavengers [22]. Many neurodegenerative and cardiovascular diseases have been linked to oxidative stress which is also a major cause for aging [23]. Lifespan assay was performed at 20 °C, an optimal growth temperature for wild-type N2 *C. elegans*. (Figure 2A, Table 2). Compared to the control group, 200 μM PHZ prolonged the mean lifespan by 18.51% (*p* < 0.01).

Since natural products with pro-longevity properties often delay the onset of AD, we conjectured if PHZ would also delay the Aβ-induced pathological behavior in *C. elegans*. Therefore, we examined the effect of PHZ on the lifespan of transgenic CL4176 worms, expressing the human Aβ_1–42_ peptide in body wall cell. In general, the Aβ toxicity leads to paralysis and premature death. Interestingly, we observed that worms treated with 200 μM PHZ exhibited the extension of mean lifespan from 7.288 to 10.122 days (Figure 2B, Table 2). Taken together, these results indicated for PHZ effects on improving longevity and delaying the onset of AD in *C. elegans*.

### 2.3. PHZ Protects Against Aβ Toxicity

Oxidative stress-induced Aβ toxicity is the potential cause of neurodegenerative diseases such as AD. Since Aβ toxicity triggers paralysis in the worm body, a paralysis assay was performed to examine the possible alterations of Aβ toxicity upon PHZ treatment. In the transgenic *C. elegans* strain CL4176 model, an increase of temperature from 16 to 25 °C induces expression of human Aβ_1–42_ in body wall muscle of *C. elegans*, resulting in an Aβ-dependent paralysis phenotype. We found that 200 μM PHZ treatment significantly affected the paralysis time of CL4176 worms by delaying the paralysis (Figure 2C, Table 2) and increasing head bends, these findings suggested that PHZ can potentially protect against Aβ-induced toxicity.

### 2.4. PHZ Reduced Lipofuscin Accumulation in CL4176

**Figure 3:**
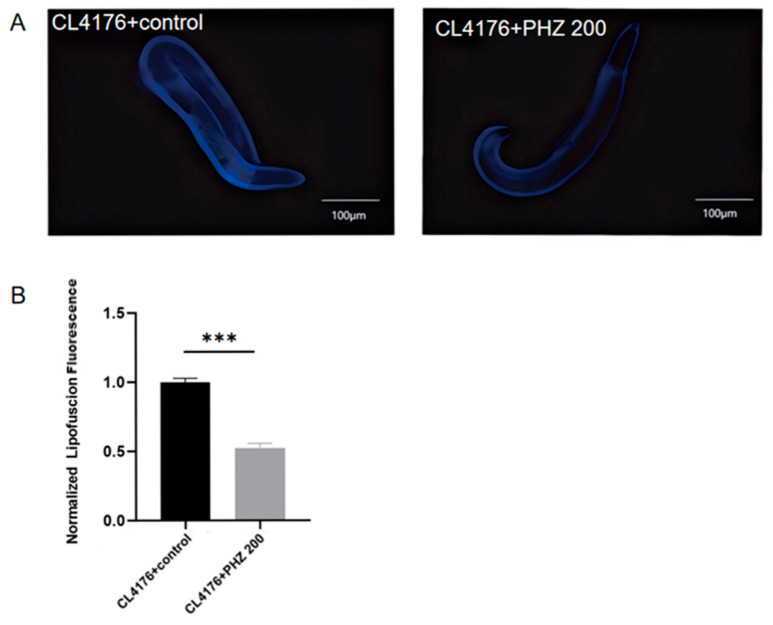
Effect of PHZ in Lipofuscin accumulation in AD model *C. elegans* with/without PHZ. (**A**) Representative fluorescent images (10×) depicting of the control group and the PHZ group. (**B**) Lipofuscin content with bar graph representing relative fluorescence intensity upon PHZ-treated worms compared to their respective control. Relative fluorescence intensity was measured using image J software (Pro Plus 6.0), *n* ≥ 20. *** (*p* < 0.001).

Clinical evidence suggests that compounds that slow aging and extend life expectancy are effective against AD [24]. Lipofuscin, a sensitive aging biomarker, accumulation diminishes autophagocytotic capacity and affects the recycling of cellular components [25]. It accumulates with age and is often used as a marker of aging, which is influenced by the content of polyunsaturated fatty acids. We checked for the accumulation of lipofuscin (Figure 3A,B), We found that lipofuscin content significantly reduced (*p* < 0.001) in PHZ-treated AD model CL4176 worms compared to control.

### 2.5. Lipidomics Reveals the Role of PHZ in Regulating Lipid Metabolism in CL4176

**Figure 4:**
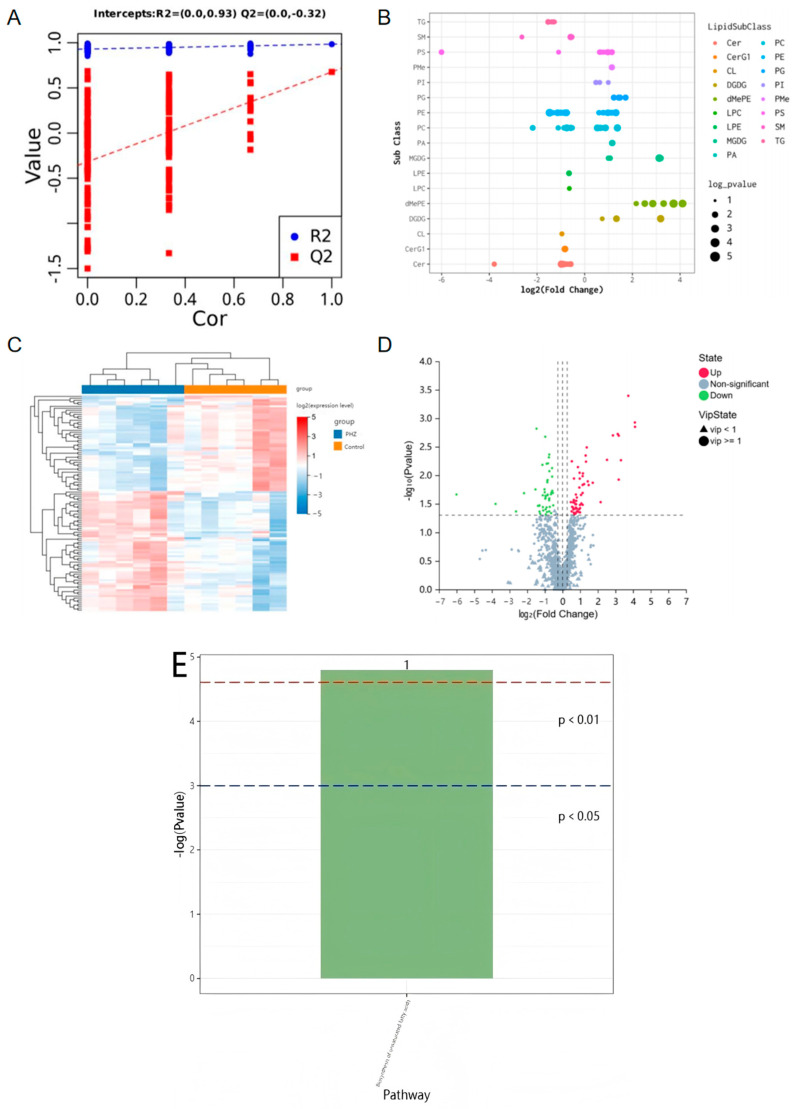
Effect of PHZ on CL4176 from the perspective of lipid metabolism. (**A**) OPLS-DA cluster analysis of nematodes under 200 μM PHZ treatment, and 200 times response permutation test (RPT, response permutation testing) of the OPLS-DA model showed no risk of overfitting. (**B**) Horizontal coordinates are log2-transformed fold change, and the vertical coordinate is the lipid subclass. Each dot in the graph represents a differential lipid, the color of the dot corresponds to a different lipid subclass, and the size of the dot is −log10-transformed p. (**C**) Each row in the graph represents a differential metabolite, each column represents a sample, and the colours represent the expression, with blue to red corresponding to low to high expression. (**D**) Horizontal coordinates are log2-converted fold change; vertical coordinates are −log10-converted q-value. Green is down-regulated significant differential metabolites, red is up-regulated significant differential metabolites; non-significant metabolites are in grey, up-regulated differential metabolites are 60, and down-regulated differential metabolites are 48. Circles are for metabolites with VIP greater than or equal to 1, triangles are for metabolites with VIP smaller than 1, and non-significant metabolites are grey. (**E**) Metabolic pathway enrichment analysis of differential metabolites based on the KEGG database, enriched in unsaturated fatty acid metabolic pathway (>1000 worms per sample, collected from six independent experiments).

It is well known that lipid metabolism is critical for longevity in *C. elegans* [26], and maintaining normal lipid metabolism ensures a longer lifespan for the worms. In addition, Aβ toxicity induced by lipid metabolism disorders is a potential cause of neurodegenerative diseases such as AD [4]. The lipid metabolism theory of aging suggests that aging is caused by disorders of lipid metabolism. Many lipid metabolism modulators have neuroprotective functions, many neurodegenerative diseases are associated with lipid metabolism disorders, and lipid metabolism disorders are also a major cause of aging. Lipid metabolism profiles of *C. elegans* treated with 200 μM PHZ were analyzed by LC-MS. OPLS-DA cluster analysis showed significant separation between groups, with a model fit R2Y value of 0.93 and a predicted Q2 value of −0.32 (Figure 4A). There were 108 potential lipid markers, of which 60 lipid subclasses were up-regulated and 48 lipid subclasses were down-regulated. Heat maps, volcano maps and specific lipid subclasses show that PHZ had a significant regulatory effect on nematode lipid metabolism (Figure 4B–D). Metabolite pathway enrichment analysis was performed using the KEGG database, and differential lipid metabolites were enriched in the unsaturated fatty acid pathway (Figure 4E).

### 2.6. PHZ Reduces Lipid Accumulation in Transgenic C. elegans CL4176

**Figure 5:**
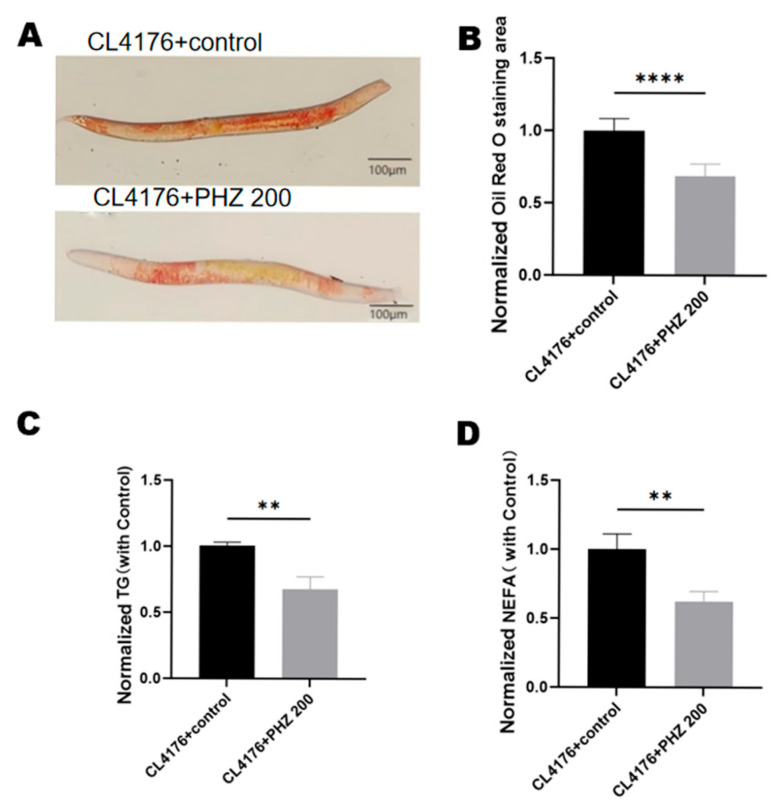
Effect of PHZ on lipid content of CL4176.CL4176 was treated with PHZ for 24 h. (**A**) Oil Red O (ORO) visualization of lipid content of different groups of CL4176; *n* ≥ 36. (**B**) Quantitative results of ORO. (**C**) Triglyceride (TG) level. (**D**) Non Esterified Fatty Acid (NEFA) level. Values are expressed as mean ± SD. (**B**–**D**) **** (*p* < 0.0001) ** (*p* < 0.01) ((**C**,**D**): >1000 worms per sample, collected from three independent experiments). TG and NEFA levels were standardized by protein content.

We investigated 200 µM PHZ on triglyceride (TG) and non-esterified fatty acid (NEFA) levels in worms. The results showed that the PHZ-treated group significantly reduced the levels of TG and NEFA in CL4176 (Figure 5C,D). TG (*p* < 0.01)and NEFA levels in *C. elegans* after treatment with 200 μM PHZ (*p* < 0.001) compared to the control group. Lipid accumulation, as characterized by ORO staining (Figure 5A), was observed to be significantly lower in the PHZ treatment group, and the lipid area was significantly less than that of the control group (*p* < 0.0001). The above experimental results confirmed that PHZ reduced lipid accumulation in CL4176.

### 2.7. qRT-PCR Results Indicate That PHZ Protects AD Worms from Aβ Toxicity and Prolongs Lifespan by Enhencing Fatty Acids β-Oxidation

**Figure 6:**
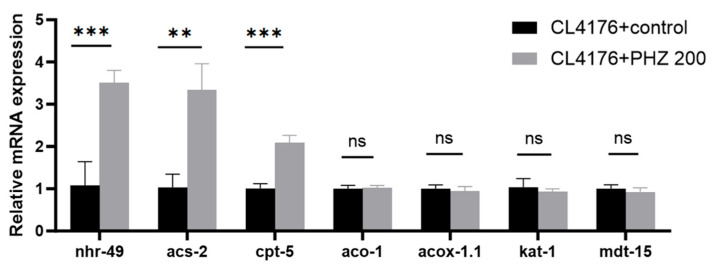
qRT-PCR results: effect of PHZ on the expression of CL4176 fatty acid βOX-related genes; data are mean ± SD (*n* = 3, from 3 independent experiments) ***, **, ns represent *p* < 0.001, *p* < 0.01, and non-significant difference.

Compared with the control group, the PHZ-treated group can significantly up-regulate the gene expression of worms’ *nhr-49* (*p* < 0.001), *acs-2* (*p* < 0.01), and *cpt-5* (*p* < 0.001), and had no significant effect on *aco-1*, *acox-1.1*, *kat-1*, and *mdt-15* (Figure 6). *nhr-49* is the upstream gene of *acs-2* and *cpt-5* [27], and is the key gene for fatty acid β-oxidative factor, and is the homolog of mammalian peroxisome proliferator-activated receptors (PPARα) [28,29]. In the process of β-oxidation, *acs-2* and *cpt-5* encode mitochondrial acyl-coenzyme A synthetase and carnitine palmitoyl transferase, respectively; qRT-PCR results suggested that the key genes on the fatty acid β-oxidation pathway, *nhr-49*, *acs-2*, and *cpt-5,* are key genes in the regulation of CL4176 lipid metabolism by PHZ.

### 2.8. Molecular Docking Results Show That NHR-49 Binds to PHZ

**Figure 7:**
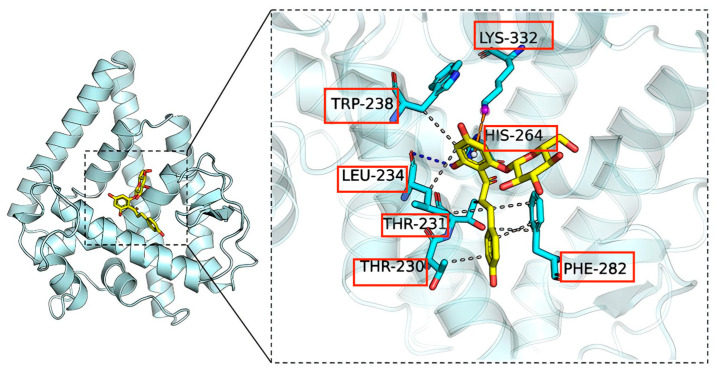
Binding pattern of NHR-49 and PHZ obtained based on docking. The left figure shows the overall view, and the right figure shows the partial view; in the figure the yellow stick is the small molecule, the cyan cartoon is the protein, the blue line indicates the hydrogen bonding interaction, the grey dotted line indicates the hydrophobic interaction, and the orange dotted line indicates the cation-pi interaction.

Docking simulation technique is a convenient and effective means to probe the interaction of small molecules with targets. Docking studies of PHZ with NHR-49 protein were performed using Vina 1.2.3 software. The interaction diagram of PHZ with NHR-49 protein is shown below (Figure 7). We can see that PHZ forms hydrogen bonding interaction with LEU-234, and HIS-264 on NHR-49 protein. The formation of hydrogen bonds results in tighter binding between the protein and PHZ. In addition, PHZ interacts with LYS-332 on NHR-49 in a cation-pi interaction, and forms hydrophobic interactions with LEU-234, TRP-238, PHE-282, THR-230, and THR-231, which provide the molecule with strong van der Waals forces. A negative binding affinity indicates the possibility of binding, and usually smaller values are considered more likely to bind. In this complex, the docking software gave PHZ a binding affinity score of −6.813 kcal/mol for NHR-49, implying that PHZ binds relatively well to the NHR-49.

### 2.9. NHR-49 Was Essential to PHZ Mediated Protection of Worms’ Lipid Metabolism

**Figure 8:**
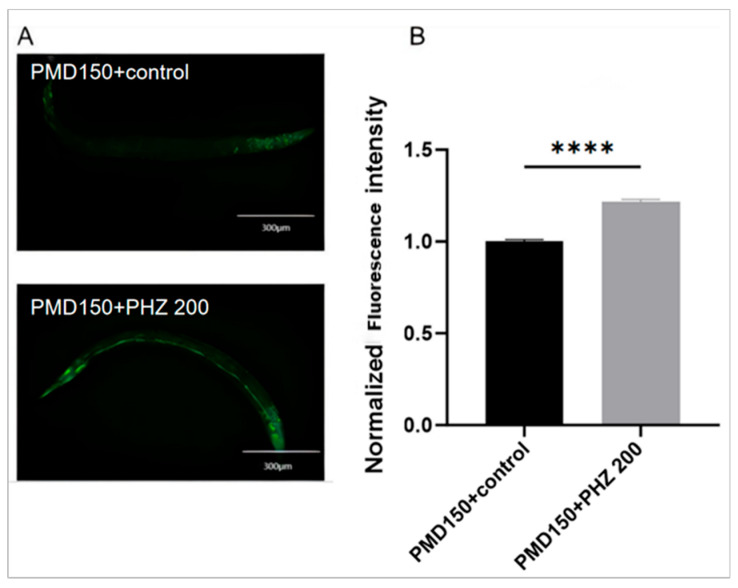

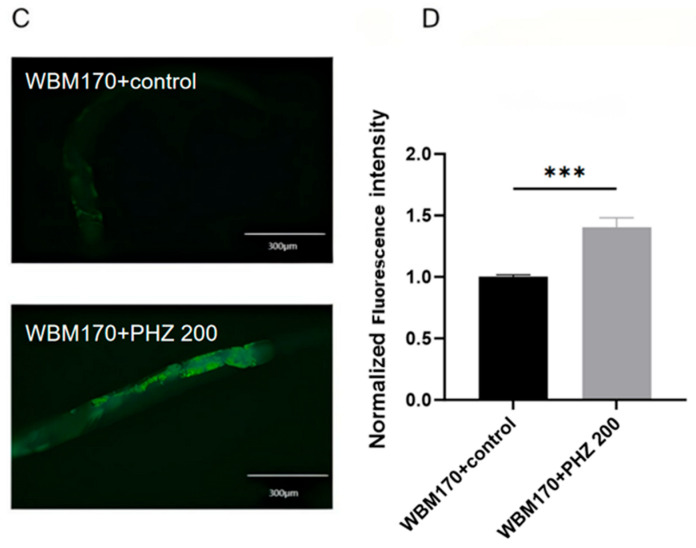
Effect of PHZ on NHR49::GFP (PMD150) and ACS::GFP (WBM170); (**A**,**C**) Worms were treated with PHZ for 24 h and then transferred to a fluorescence microscope for fluorescent protein expression assay; representative photos are shown in (**B**,**D**). Compared with the control group, NHR-49::GFP and ACS-2::GFP were expressed at a higher level in the nematodes after PHZ administration. *n* = 20, images were analyzed using ImageJ software, and numerical data were subjected to Student’s t-test using Graphpad Prism 10 software. *** (*p* < 0.001) **** (*p* < 0.0001) Values are expressed as mean ± SD.

To further explore the functional contributions of NHR-49 and ACS-2 in PHZ-mediated enhancement of fatty acid β-oxidation and attenuation of Aβ toxicity, we performed reporter-based expression analyses using transgenic *C. elegans* strains. Specifically, we utilized PMD150 (carring NHR-49::GFP) and WBM170 (carrying ACS-2::GFP) as transcriptional biosensors. GFP fluorescence analysis demonstrated significantly increased expression of both NHR-49::GFP (PMD150 strain) and ACS-2::GFP (WBM170 strain) in PHZ-treated worms relative to controls (Figure 8). These observations corroborate our preceding qRT-PCR data and collectively indicate that PHZ treatment alleviates Aβ toxicity and enhances fatty acid β-oxidation by upregulating NHR-49 and ACS-2 expression.

**Figure 9:**
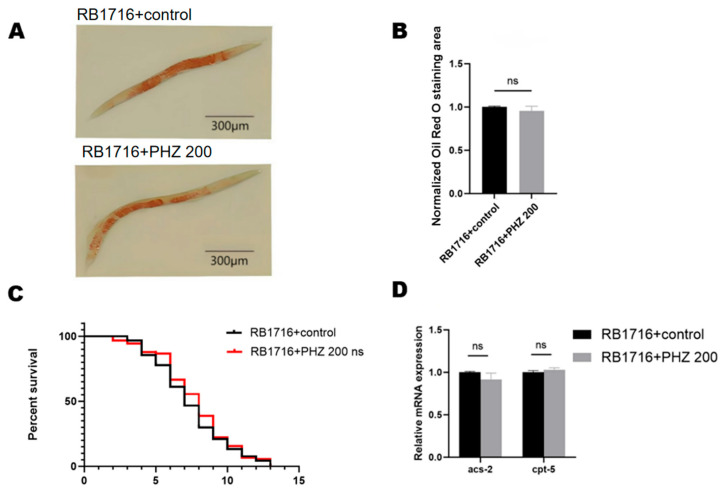
PHZ has no effect on lipid levels and lifespan and downstream gene expression in *nhr-49* mutant. (**A**) Representative image of ORO of *nhr-49* deletion mutant (RB1716). (**B**) Quantitative results of ImageJ analysis of ORO showed no significant difference. (**C**) PHZ has no significant effect on lifespan of RB1716. (**D**) PHZ has no effect on expression of downstream gene *acs-2* and *cpt-5*; ns represents non-significant difference.

Meanwhile, we performed lifespan assay in an *nhr-49* loss-of-function mutant (RB1716), and the experimental results showed that PHZ failed to prolong the lifespan of *nhr-49* mutant nematodes (Figure 9C) or reduce their lipid content (Figure 9A,B), which suggests that *nhr-49* is essential for PHZ-mediated prolongation of lifespan and regulation of lipid metabolism. qRT-PCR assay of RB1716 showed that there was also no significant difference in *acs-2* and *cpt-5* content after PHZ treatment (Figure 9D), further verifying that PHZ activates the *nhr-49* gene and thus the downstream fatty acid β-oxidation-related genes. These results indicated that NHR-49 was required for the effect of PHZ on fatty-acid β-oxidation and anti-oxidative stress. At least in part, PHZ protected AD worms against Aβ toxicity due to activate NHR-49.

## 3. Discussion

**Figure 10:**
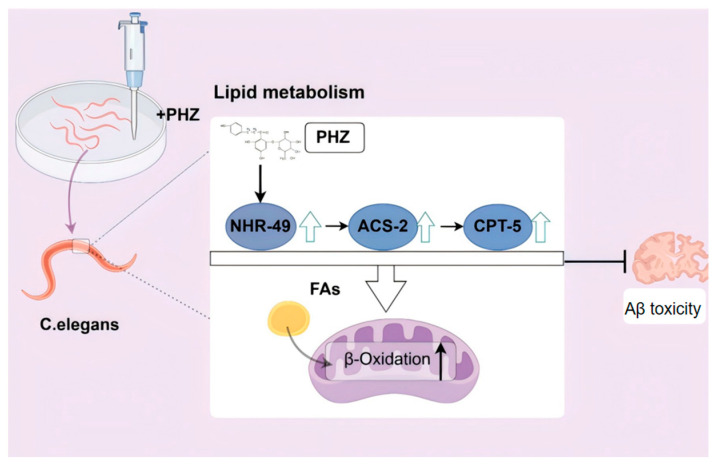
PHZ regulates lipid metabolism disorders and alleviates Aβ toxicity by promoting the expression of NHR-49 in AD worms, which in turn promotes the expression of ACS-2 and CPT-5.

This study elucidates the neuroprotective effects of PHZ and its molecular mechanisms using *C. elegans* AD models, revealing that this compound mitigates β-amyloid (Aβ) toxicity through lipid metabolism regulation (Figure 10). Initial acute thermal stress assays in wild-type N2 nematodes established 200 μ M as the optimal PHZ concentration, which significantly enhanced stress resistance and extended lifespan. Given the established correlation between longevity-promoting compounds and delayed AD onset [10], we further validated PHZ in the Aβ-transgenic CL4176 strain [30], a well-established AD model. PHZ treatment not only improved stress response capacity but also remarkably extended the mean lifespan, suggesting its potential to modulate AD pathology through conserved aging-related pathways.

The emerging “dual-engine” hypothesis posits lipid metabolic dysregulation as both a pathological contributor and Aβ toxicity amplifier in AD [31]. Our multidimensional analyses demonstrated PHZ’s synergistic modulation of these interconnected pathological features: (1). Lipidomic profiling revealed PHZ-induced metabolic remodeling in CL4176 worms, corroborated by Oil Red O (ORO) staining and quantitative assays showing significant reductions in lipid deposition (TGs and NEFAs); (2). Notably, this metabolic improvement strongly correlated with neuroprotection—PHZ reduced Aβ-induced paralysis. This metabolic–neuroprotective synergy parallels hesperidin’s mechanism in murine AD models [32], suggesting lipid metabolic modulation as a common target for neuroprotective agents. Subsequent pathway analysis indicated decreased fatty acid levels in PHZ-treated worms, implicating enhanced β-oxidation as a key mechanism.

To dissect PHZ’s molecular targets in lipid regulation, we systematically analyzed fatty acid β-oxidation-related genes. qRT-PCR results showed that PHZ had no significant effect on the expression of *aco-1*, *acox-1.1*, *kat-1*, or *mdt-15*. *aco-1* participates in the tricarboxylic acid (TCA) cycle. Its human ortholog, aconitase 1 (ACO-1), is associated with Alzheimer’s disease (AD) and functions as a key metabolic enzyme in the TCA cycle, catalyzing the isomerization between citrate and isocitrate [33]. ACO-1 is not only a core component of metabolic pathways but is also closely linked to neurodegenerative diseases [34]. *acox-1.1* is the homolog of human acyl-CoA oxidase 1 (ACOX-1), and its encoded protein participates in the initial steps of fatty acid β-oxidation [35]. *kat-1* is mitochondrial-localized and involved in fatty acid β-oxidation; it is the homolog of human acetyl-CoA acetyltransferase 1 (ACAT-1). ACAT-1 activity may indirectly regulate fatty acid content by modulating lipid droplets, thereby influencing oxidation rates [36]. *mdt-15* encodes a mediator complex subunit that interacts with NHR-49 and acts as a coactivator for mammalian sterol regulatory element-binding protein (SREBP) to regulate lipid accumulation in vivo. However, since none of these genes exhibited significant alterations upon PHZ treatment, they were not identified as mediators of PHZ’s lipid-lowering effects [37].

However, PHZ specifically upregulated *nhr-49* (a functional analog of mammalian PPARα), *acs-2* (acyl-CoA synthetase), and *cpt-5* (carnitine palmitoyl transferase), with corresponding increases in NHR-49::GFP and ACS-2::GFP fluorescence intensities in transgenic strains. These findings align with mammalian studies where PPARα—a master regulator of CNS fatty acid oxidation [38]—induces expression of ACSL(ACS-2 homolog) and CPT1A (CPT-5 homolog) to promote acyl-CoA synthesis and mitochondrial fatty acid transport [39]. The observed PPARα pathway downregulation in AD patients [40] and therapeutic potential of PPAR agonists [41] further support our conclusion that PHZ exerts neuroprotection via NHR-49/PPARα activation, consistent with reported mechanisms of PPARα agonists in AD models [42,43,44].

Critical validation experiments in *nhr-49* loss-of-function mutants (RB1716) demonstrated complete abolition of PHZ-induced lipid clearance and lifespan extension. Molecular docking confirmed high-affinity PHZ binding to the NHR-49 ligand-binding domain (LBD) (binding energy: −6.813 kcal/mol), while qRT-PCR revealed abolished *acs-2*/*cpt-5* induction in mutants, establishing the NHR-49/ACS-2/CPT-5 as PHZ’s core signaling pathway. Although mammalian PPARα exhibits cell-type-specific functions in neurons [43,45] and astrocytes [46], our work provides the first evidence in *C. elegans* that PHZ-mediated neuroprotection operates through evolutionarily conserved NHR-49-driven β-oxidation.

While this study represents a significant advance, it was conducted solely in Caenorhabditis elegans. Subsequent validation of PHZ’s efficacy and safety in mammalian models remains necessary. Our next phase of research will investigate the potential cell-type-specific effects of PHZ in the mammalian brain, particularly its influence on neuron–glia metabolic coupling. Existing research indicates that neurons are highly dependent on mitochondrial energy supply. As reported in Nature Metabolism, acylcarnitines—intermediate metabolites accumulating due to impaired fatty acid β-oxidation—serve as critical lipid mediators by which reactive astrocytes cause neuronal damage. Future studies could combine mammalian and cellular models to elucidate the differential regulation of lipid metabolism by PHZ in neurons versus astrocytes and evaluate its translational potential. Furthermore, the long-term safety profile of PHZ administration in mammals warrants further investigation.

The novelty of this study lies in being the first to establish a direct link between the PHZ-NHR-49 fatty acid β-oxidation axis and alleviation of Aβ toxicity, providing a novel therapeutic target for metabolic intervention in AD. Furthermore, whether PHZ’s lipid metabolic improvements synergistically enhance Aβ clearance through mechanisms like lipotoxicity reduction requires further validation. Collectively, this work provides critical theoretical foundations for developing AD treatment strategies based on lipid metabolic reprogramming.

## 4. Materials and Methods

### 4.1. Strains and Maintenance

*C. elegans* strains N2-Bristol (wild type), CL4176, PMD150, WBM170, and RB1716 and Escherichia coli OP50 (OP50) were purchased from the Caenorhabditis Genetics Center (CGC) (Table 3), University of Minnesota, MN, USA. The strains were grown and maintained under standard laboratory conditions at 20 °C or 16 °C on nematode growth medium (NGM) agar plates seeded with a lawn of OP50. Phlorizin (purity ≥ 98%, molecular weight 436.41) was purchased from Shanghai Yuanye Biotechnology Co. (Shanghai, China).

OP50 Seeding:

Within a biosafety cabinet, pipette 200 μL of OP50 bacterial suspension onto the center of NGM plates. Allow the suspension to form a circular lawn without contacting the plate edges to prevent *C. elegans* from crawling to the periphery and desiccating. After 12 h of drying at room temperature, bacterial lawns are ready for use.

PHZ Administration:

Dissolve phlorizin in deionized water via heating and sonication, then filter-steri lize (0.22 μm) to prepare a 2 mmol/L stock solution. Store protected from light at 4 °C. For treatment, mix the stock solution with *E. coli* OP50 at varying ratios to achieve desired concentrations. Apply 200 μL of the drug-bacterial mixture onto NGM plates. Following 12 h of drying, transfer synchronized worms onto drug-containing plates for culturing.

### 4.2. Synchronization

Hypochlorite bleaching method: Culture plates containing abundant gravid adults C.elegans were selected. Approximately 1–2 mL of M9 buffer was used to wash the plates, and the M9 buffer containing worms was collected into fresh centrifuge tubes. Samples were centrifuged at 1200 rpm for 1 min to pellet the worms. The supernatant was discarded, and the pellet was washed 2–3 times with M9 buffer. After the final wash, 1 mL M9 buffer was retained in the tube. Subsequently, 1.5 mL of bleaching solution was added with continuous vortexing until complete dissolution of adult worm bodies was observed while eggs remained intact. Tubes were centrifuged at 1200 rpm for 2 min, and the supernatant was discarded, leaving the egg pellet at the bottom. The pellet was washed 2–3 times with M9 buffer to remove residual bleach and prevent damage to hatched larvae. Washed eggs were resuspended in M9 buffer and incubated in a constant-temperature incubator. Newly hatched larvae remained arrested at the L1 stage due to food deprivation. L1 larvae were pelleted by centrifugation at 3000 rpm for 2 min, resuspended, and transferred to OP50-seeded NGM plates. After culturing to the L4 stage followed by an additional 24-h growth period, synchronized populations were obtained for subsequent experiments.

### 4.3. Lifespan Assay

N2 and RB1716 lifespan assays were performed at 20 °C. CL4176 worms were grown at 16 °C until they reached the L4 stage and then transferred to 20 °C. After the L4 stage, 30 worms were transferred to fresh NGM (PHZ or *E. coli* OP50 added) per plate, and survival was assessed daily until the last worm was dead, A worm was considered dead if it showed no response to gentle touch, experiments were repeated at least 3 times.

### 4.4. Paralysis Assay

CL4176 age-synchronized eggs were cultured to the L4 larval stage on NGM plates supplemented with either OP50 or 200 μM PHZ for 24 h. Aβ expression was subsequently induced by shifting the incubation temperature from 16 °C to 25 °C. Paralysis was defined as the absence of full-body movement or restricted head thrashing upon gentle mechanical stimulation. At least 90 worms were used in each group. Groups were monitored daily until complete paralysis was observed.

### 4.5. Resistance Assay

For acute heat stress analysis, after different treatments, synchronized N2 and CL4176 nematodes were subjected to 35 °C in a temperature-controlled incubator. Cohorts of ≥90 worms per experimental group were sampled at 2-h intervals for viability assessment. Mortality scoring was performed by (1) confirming cessation of pharyngeal pumping, and (2) absence of movement response to anterior/posterior tactile stimulation using a platinum wire pick. Specimens displaying desiccation artifacts (adherence to plate walls) were excluded from quantification. The assay continued until complete cohort mortality (0% survival) on NGM plates was achieved.

For the paraquat-induced stress resistance assay, antioxidant activity was measured in *C. elegans* as previously described [47]. A 1 M paraquat stock solution was prepared by dissolving 1 g paraquat in 3.89 mL H_2_O and stored at 4 °C. Separately, 1 g FUdR was dissolved in 40.62 mL H_2_O (not autoclaved) and stored at −80 °C. For assay plates, 1 M paraquat stock was diluted in liquid NGM medium to 4 mM final concentration, followed by addition of FUdR to 100 μM final concentration with thorough mixing. The mixture was poured into plates and solidified at room temperature. *E. coli* OP50 or 200 µM PHZ was seeded onto the solidified plates. Thirty synchronized L4-stage CL4176 worms were transferred to each paraquat-containing plate. We monitored worm survival daily, recording live/dead status and censoring individuals lost (e.g., desiccated on plate walls). The experiment was independently repeated three times.

### 4.6. Reactive Oxygen Species (ROS) Assay

Intracellular ROS levels in *C. elegans* were detected using the fluorescent probe 2′,7′-dichlorodihydrofluorescein diacetate (DCFH-DA) [8]. Synchronized CL4176 worms at the L4 stage were transferred to plates containing the respective treatments. Following 24 h of different exposure at 16 °C, worms were subjected to acute thermal stress at 35 °C for 30 min. Worms were then collected from the plates into 200 µL microtubes using M9 buffer and washed three times with M9 buffer. Subsequently, 100 µL of 1 mM DCFH-DA solution was added to the worms. Tubes were wrapped in aluminum foil and incubated in the dark at 37 °C for 30 min. After incubation, worms were washed three times with M9 buffer and anesthetized with 5 mM levamisole for 1 min. The anesthetized worms were transferred onto a 2% agarose pad and covered with a coverslip. Worms were visualized and imaged under a fluorescence microscope. Fluorescence intensity within the worms was quantified using ImageJ software. Data are presented as bar graphs (*n* = 20 worms per group).

### 4.7. Head-Bending Assay

Briefly, synchronized CL4176 *C. elegans* were treated with or without 200 µM PHZ for 24 h, and ≥36 CL4176 nematodes per group were individually transferred to NGM plates using a picker. After 30 s acclimatization, movements were quantified by counting lateral deflections from the body’s longitudinal axis during a 30 s observation window.

### 4.8. Lipofuscin Assay

Synchronized L4 stage CL4176 worms were treated with or without 200 µM PHZ for 8 days. Images were captured using a fluorescence microscope (Nikon, Tokyo, Japan). Twenty worms were measured and detected with blue fluorescence (Ex/Em 350/460 nm) [48]. The blue fluorescence was measured with ImageJ, and the background signal was subtracted.

### 4.9. Triglyceride and NEFA Quantification

Synchronized L4-stage CL4176 worms were treated with or without 200 µM PHZ for 24 h. After the excessive *E. coli* OP50 were completely washed with M9 buffer, the CL4176 nematodes were transferred and crushed with a glass homogenizer within a tube in an ice bath. The suspension was centrifuged according to the instructions of the kit (Nanjing Jiancheng Institute of Bioengineering, Nanjing, China), and the supernatant was extracted to measure TG and NEFA. The protein concentration was determined with the BCA kit (Thermo Scientific™, Waltham, MA, USA) to standardize the activity of TG and NEFA levels [49].

### 4.10. Oil Red O Assay

The fat accumulation of *C. elegans* was detected by ORO staining according to the described protocol [50]. Synchronized L4-stage CL4176 worms were treated with or without 200 µM PHZ for 24 h. The nematodes were washed with M9 buffer. The ORO stock solution was diluted with deionized water (ORO stock solution: deionized water = 6:4, *v*/*v*) and filtrated by 0.45 µM filter to remove impurities. After that, 500 µL of 60% isopropanol was used to dehydrate and fix the nematodes. Subsequently, centrifugation was applied to obtain the nematodes which were stained with 500 µL of ORO solution for 2 h. The stained nematodes were washed with M9 buffer and then photographed by a microscopy. A total 36 nematodes were photographed in each experimental group. Images were analyzed using Image J software.

### 4.11. qRT-PCR Experiments

Synchronized L4-stage CL4176 and RB1716 worms were treated with or without 200 µM PHZ for 24 h; the worms were than collected and washed with M9 buffer. Total RNA was extracted with a Solarbio kit. Reverse transcription was performed with a Thermo Fisher RT kit. The total system of fluorescence quantitative PCR reaction was 20 µL, and the lysis curve and amplification curve were analyzed at the end of the reaction, and the gene expression level was analyzed by the relative quantitative method 2^−ΔΔCT^, and normalized to the gene *β-actin* (Table 4). The primers were synthesized and ordered by Shanghai Bioengineering.

### 4.12. Fluorescence Detection Experiments

Synchronized L4 stage PMD150 and WBM170 worms were treated with or without 200 µM PHZ for 24 h; the worms were then collected and washed with M9 buffer. Protein expression associated with green fluorescent protein (GFP) strains was measured by fluorescence imaging as previously described using PMD150, and WBM170. Worms paralyzed using 5 mM levamisole were placed on slides containing pads with 2% agarose. Photographs were taken and data collected using a Nikon fluorescence microscope the images were analyzed with the ImageJ software and numerical data were analyzed by Student’s t-test using Graphpad Prism 10 software.

### 4.13. Lipidomics Assay

Synchronized L4 stage CL4176 and RB1716 worms were treated with or without 200 µM PHZ for 24 h; the worms were then collected and washed with M9 buffer. A Waters UPLC I-Class Plus (Waters, Milford, MA, USA) coupled with a Q Exactive high-resolution mass spectrometer (Thermo Fisher Scientific, Waltham, MA, USA) was used for metabolite separation and detection. Spectrometric conditions: The column used was a CSH C18 column (1.7 µm 2.1 × 100 mm, Waters, USA). The positive separation mode mobile phases were 60% plutonitrile water + 10 mM ammonium formate + 0.1% formic acid (Liquid A) and 90% isopropanol + 10% plutonitrile + 10 mM ammonium formate + 0.1% formic acid (Liquid B), while the negative separation mode mobile phases were 60% plutonitrile water + 10 mM ammonium formate (Liquid A) and 90% isopropanol + 10% plutonitrile + 10 mM ammonium formate (Liquid B). The elution was performed using the following gradient: 0~2 min, 40~43% B solution; 2~2.1 min, 43~50% B solution; 2.1~7 min, 50~54% B solution; 7~7.1 min, 54~70% B solution; 7.1~13 min, 70~99% B solution; 13~13.1 min, 99~40% B solution; 13.1~15 min, 40% B solution; 13.1~15 min, 40% B solution. ~15 min, 40% B liquid. The flow rate was 0.4 mL/min, the column temperature was 55 °C, and the injection volume was 5 µL.

Mass spectrometry (MS): One-stage and two-stage mass spectrometry data acquisition was performed using a Q Exactive mass spectrometer (Thermo Fisher Scientific, USA). The mass ratio of mass spectrometry was 200–2000, the resolution of the first stage was 70,000, the AGC was 3 × 10^6^, and the maximum injection time (IT) was 100 ms. The Top3 was selected for fragmentation according to the intensity of mothers’ isolation, and the information of the second stage was collected with the resolution of the second stage of 17,500, the AGC was 1 × 10^5^, and the maximum injection time (IT) was 50 ms. The second stage resolution was 17,500, the AGC was 1 × 10^5^, the maximum injection time (IT) was 50 ms, and the stepped nce was set to 15, 30, and 45 eV. The ESI parameters were set to 40 for the sheath gas flow rate, 10 for the auxiliary gas flow rate, and 10 for the spray voltage (|KV|). The spray voltage (|KV|) was 3.80 for the positive sub-mode and 3.20 for the negative sub-mode. The capillary temp was 320 °C, and the auxiliary gas heater temp was 350 °C. The temperature was set at 320 °C for the sub-tube and 350 °C for the auxiliary gas heater.

### 4.14. Molecular Docking

The crystal structure of the NHR-49 protein used for docking was obtained from the PDB database by downloading the PDB ID of NHR-49 as 3VI8, and the 3D structure of the small molecule phlorizin was obtained from the PUBCHEM database by downloading the PUBCHEM database and energy-minimized under the MMFF94 force field. AutoDock Vina 1.2.3 software was used for molecular docking work, and before docking began, the receptor proteins were treated using PyMol 2.5.5, which included the removal of water molecules, salt ions, and small molecules. Subsequently, a docking box was set up with the crystal ligand center in the center of the box, and the size of the box was 30 × 30 × 30 cubic angstroms. In addition, all treated small molecules as well as receptor proteins were converted to the PDBQT format necessary for AutoDock Vina 1.2.3 docking using ADFRsuite 1.0. When docking, the exhaustiveness of the global search was set to 32, and the rest of the parameters were left at their default settings. The output docked conformations with the highest scores were considered to be the binding conformations and finally the docking results were visualized and analyzed using PyMol 2.5.5.

### 4.15. Statistical Analysis

All data were expressed as mean ± SD. In the lifespan assay, the significance of comparison was calculated by the log-rank test, while the others were compared using the Student’s t-test unless mentioned otherwise. Statistical analysis was performed with the GraphPad Prism 10 (GraphPad Software, San Diego, USA). *p* < 0.05 was considered statistically significant (* *p* < 0.05, ** *p* < 0.01, *** *p* < 0.001, **** *p* < 0.0001). Each of the assays was independently repeated at least thrice and the corresponding data are shown.

## 5. Conclusions

PHZ attenuates Aβ toxicity and lipid accumulation in AD worms by enhancing fatty acid β-oxidation via the NHR-49/ACS-2/CPT-5, demonstrating dual neuroprotective and metabolic regulatory efficacy. Collectively, this study systematically demonstrates that PHZ ameliorates AD pathology by targeting lipid metabolism, thereby exploring new avenues for anti-AD drug development while lending theoretical support for lipid metabolic intervention in AD.

## Figures and Tables

**Table 1:** Heat stress analysis: the effect of various concentrations of PHZ on the mean survival time (hours) of wild-type (N2) and AD model (CL4176).

*C. elegans* Model	Treatment	Mean	±SD	%	*p* Value (Log-Rank Significance)
N2	0 µM	5.922	0.81		
	100 µM	6.633	0.24	12.01	ns
	200 µM	7.878	0.93	33.03	*p* < 0.0001
	500 µM	5.333	0.38	−9.95	*p* < 0.05
	700 µM	5.022	0.56	−15.20	*p* < 0.01
CL4176	0 µM	7.8	0.82		
	100 µM	8.056	0.57	3.28	ns
	200 µM	8.756	1.32	12.26	*p* < 0.01
	500 µM	7.122	0.52	−8.69	*p* < 0.05
	700 µM	6.122	1.04	−21.51	*p* < 0.0001

**Table 2:** Survival analysis of paralysis, paraquat and lifespan.

*C. elegans* Model	Treatment	Mean	±SD	%	*p* Value (Log-Rank Significance)
N2 (lifespan)	0 µM	9.722	0.79		
	200 µM	11.522	1.32	18.51	*p* < 0.01
CL4176 (lifespan)	0 µM	7.288	1.08		
	200 µM	10.122	0.71	38.89	*p* < 0.0001
CL4176 (paralysis)	0 µM	4.322	0.28		
	200 µM	5.377	0.91	24.41	*p* < 0.01
CL4176 (paraquat)	0 µM	5.7	1.03		
	200 µM	6.433	0.89	12.86	*p* < 0.0001

**Table 3:** *C. elegans* strains.

*C. elegans*	Relevant Genetic Background	Characteristics
N2	Wild type	Applied in aging, basic biology with high fecundity
CL4176	dvIs27 [myo-3p::A-Beta (1-42)::let-8513’UTR) + rol-6 (su1006)]	Applied in Alzheimer’s disease mechanisms and drug screening, temperature-induced paralysis
PMD150	utsIs4 [nhr-49p::nhr-49::GFP + myo-2p::mCherry]	NHR-49::GFP; green fluorescent protein-tagged NHR-49
WBM170	wbmEx57 [acs-2p::GFP + rol-6 (su1006)]. Pick Rollers to maintain.	ACS-2::GFP; green fluorescent protein-tagged ACS-2
RB1716	nhr-49 (ok2165) loss-of-function mutation	Increased lipid deposition, β-oxidation defects

**Table 4:** The primers involved in qRT-PCR.

Gene	Seqence Forward (5′–3′)	Seqence Reverse (5′–3′)
*nhr-49*	CAGATGACGCACCCACAAGATATG	GAATGAACTCGGAGAGCAGAGAATC
*acs-2*	GCAGCCTCGCTCTACACTCT	GACTCCTGCAAATGCACATGC
*cpt-5*	GCTCGGCGTGCTCCATATCATC	TCAAGATTCGGCAACGGAAGACG
*aco-1*	GATGGAAGTGGTGTTCTTGGATGG	AGTCGGTACTGGTAACGGTATCAC
*acox-1.1*	TTCAACAACTACCGTATCCCAAGAAC	AATCGCCTGTCCAGTAAGCATATAAC
*kat-1*	CCGCCACGCACCCACTC	CATTAACTTCCCATTGAGCAACATCTG
*mdt-15*	CTCCAGATCCACAACCAACATCAG	GCGGCAACAGCAGCAGTG
*β-actin*	GCCGGAGACGACGCTCCACGCG	GCCTCGTCTCCGACGTACGAGTC

## Data Availability

The original contributions presented in this study are included in the article. Further inquiries can be directed to the corresponding authors.

## References

[1] Wurtman R. (2015). Biomarkers in the diagnosis and management of Alzheimer’s disease. Metab. Clin. Exp..

[2] Reiss A.B., Arain H.A., Stecker M.M., Siegart N.M., Kasselman L.J. (2018). Amyloid toxicity in Alzheimer’s disease. Rev. Neurosci..

[3] Ooi K.M., Vacy K., Boon W.C. (2021). Fatty acids and beyond: Age and Alzheimer’s disease related changes in lipids reveal the neuro-nutraceutical potential of lipids in cognition. Neurochem. Int..

[4] Tong B., Ba Y., Li Z., Yang C., Su K., Qi H., Zhang D., Liu X., Wu Y., Chen Y. (2024). Targeting dysregulated lipid metabolism for the treatment of Alzheimer’s disease and Parkinson’s disease: Current advancements and future prospects. Neurobiol. Dis..

[5] Bogie J.F.J., Haidar M., Kooij G., Hendriks J.J.A. (2020). Fatty acid metabolism in the progression and resolution of CNS disorders. Adv. Drug Deliv. Rev..

[6] Burghardt K.J., Kajy M., Ward K.M., Burghardt P.R. (2023). Metabolomics, Lipidomics, and Antipsychotics: A Systematic Review. Biomedicines.

[7] Biessels G.J., Strachan M.W., Visseren F.L., Kappelle L.J., Whitmer R.A. (2014). Dementia and cognitive decline in type 2 diabetes and prediabetic stages: Towards targeted interventions. Lancet Diabetes Endocrinol..

[8] Nilsson M.I., Tarnopolsky M.A. (2019). Mitochondria and Aging-The Role of Exercise as a Countermeasure. Biology.

[9] Shin S.K., Cho S.J., Jung U.J., Ryu R., Choi M.S. (2016). Phlorizin Supplementation Attenuates Obesity, Inflammation, and Hyperglycemia in Diet-Induced Obese Mice Fed a High-Fat Diet. Nutrients.

[10] Kumar S., Akhila P.V., Suchiang K. (2023). Hesperidin ameliorates Amyloid-β toxicity and enhances oxidative stress resistance and lifespan of Caenorhabditis elegans through acr-16 mediated activation of the autophagy pathway. Free Radic. Biol. Med..

[11] Ehrenkranz J.R., Lewis N.G., Kahn C.R., Roth J. (2005). Phlorizin: A review. Diabetes/Metab. Res. Rev..

[12] Zhang X.Y., Chen J., Yi K., Peng L., Xie J., Gou X., Peng T., Tang L. (2020). Phlorizin ameliorates obesity-associated endotoxemia and insulin resistance in high-fat diet-fed mice by targeting the gut microbiota and intestinal barrier integrity. Gut Microbes.

[13] Zhang S., Wang X., Liu S., Hu C., Meng Y. (2025). Phlorizin ameliorates cognitive and behavioral impairments via the microbiota-gut-brain axis in high-fat and high-fructose diet-induced obese male mice. Brain Behav. Immun..

[14] Wright E.M. (2021). SGLT2 Inhibitors: Physiology and Pharmacology. Kidney360.

[15] Mei X., Li Y., Zhang X., Zhai X., Yang Y., Li Z., Li L. (2024). Maternal Phlorizin Intake Protects Offspring from Maternal Obesity-Induced Metabolic Disorders in Mice via Targeting Gut Microbiota to Activate the SCFA-GPR43 Pathway. J. Agric. Food Chem..

[16] Kamdi S.P., Raval A., Nakhate K.T. (2021). Phloridzin attenuates lipopolysaccharide-induced cognitive impairment via antioxidant, anti-inflammatory and neuromodulatory activities. Cytokine.

[17] Gu Q., Wang C., Huang H., Wei D., Fu L., Liu G., Zhou Q., Yang J., Fu Y. (2025). Phlorizin mitigates high glucose-induced metabolic disorders through the IIS pathway in Caenorhabditis elegans. Food Funct..

[18] Zhao H., Zhai B.W., Zhang M.Y., Huang H., Zhu H.L., Yang H., Ni H.Y., Fu Y.J. (2024). Phlorizin from *Lithocarpus litseifolius* [Hance] Chun ameliorates FFA-induced insulin resistance by regulating AMPK/PI3K/AKT signaling pathway. Phytomed. Int. J. Phytother. Phytopharm..

[19] Kumsta C., Chang J.T., Schmalz J., Hansen M. (2017). Hormetic heat stress and HSF-1 induce autophagy to improve survival and proteostasis in *C. elegans*. Nat. Commun..

[20] Wan Q.L., Meng X., Dai W., Luo Z., Wang C., Fu X., Yang J., Ye Q., Zhou Q. (2021). N(6)-methyldeoxyadenine and histone methylation mediate transgenerational survival advantages induced by hormetic heat stress. Sci. Adv..

[21] Viña J. (2019). The free radical theory of frailty: Mechanisms and opportunities for interventions to promote successful aging. Free Radic. Biol. Med..

[22] Salazar N., González S., Nogacka A.M., Rios-Covián D., Arboleya S., Gueimonde M., Reyes-Gavilán C.G.L. (2020). Microbiome: Effects of Ageing and Diet. Curr. Issues Mol. Biol..

[23] Link P., Roth K., Sporer F., Wink M. (2016). Carlina acaulis Exhibits Antioxidant Activity and Counteracts Aβ Toxicity in *Caenorhabditis elegans*. Molecules.

[24] Liu Y., Tan Y., Zhang Z., Yi M., Zhu L., Peng W. (2024). The interaction between ageing and Alzheimer’s disease: Insights from the hallmarks of ageing. Transl. Neurodegener..

[25] Zhu F.D., Chen X., Yu L., Hu M.L., Pan Y.R., Qin D.L., Wu J.M., Li L., Law B.Y., Wong V.K. (2023). Targeting autophagy to discover the *Piper wallichii* petroleum ether fraction exhibiting antiaging and anti-Alzheimer’s disease effects in *Caenorhabditis elegans*. Phytomed. Int. J. Phytother. Phytopharm..

[26] Johnson A.A., Stolzing A. (2019). The role of lipid metabolism in aging, lifespan regulation, and age-related disease. Aging Cell.

[27] Watterson A., Tatge L., Wajahat N., Arneaud S.L.B., Solano Fonseca R., Beheshti S.T., Metang P., Mihelakis M., Zuurbier K.R., Corley C.D. (2022). Intracellular lipid surveillance by small G protein geranylgeranylation. Nature.

[28] Luo R., Su L.Y., Li G., Yang J., Liu Q., Yang L.X., Zhang D.F., Zhou H., Xu M., Fan Y. (2020). Activation of PPARA-mediated autophagy reduces Alzheimer disease-like pathology and cognitive decline in a murine model. Autophagy.

[29] Wang Y.X. (2010). PPARs: Diverse regulators in energy metabolism and metabolic diseases. Cell Res..

[30] Paul D., Chipurupalli S., Justin A., Raja K., Mohankumar S.K. (2020). *Caenorhabditis elegans* as a possible model to screen anti-Alzheimer’s therapeutics. J. Pharmacol. Toxicol. Methods.

[31] Ferré-González L., Lloret A., Cháfer-Pericás C. (2023). Systematic review of brain and blood lipidomics in Alzheimer’s disease mouse models. Prog. Lipid Res..

[32] Javed H., Vaibhav K., Ahmed M.E., Khan A., Tabassum R., Islam F., Safhi M.M., Islam F. (2015). Effect of hesperidin on neurobehavioral, neuroinflammation, oxidative stress and lipid alteration in intracerebroventricular streptozotocin induced cognitive impairment in mice. J. Neurol. Sci..

[33] Shen T., Wang H., Tang B., Zhu G., Wang X. (2023). The impact of RNA binding proteins and the associated long non-coding RNAs in the TCA cycle on cancer pathogenesis. RNA Biol..

[34] Berry T., Abohamza E., Moustafa A.A. (2020). A disease-modifying treatment for Alzheimer’s disease: Focus on the trans-sulfuration pathway. Rev. Neurosci..

[35] Xue Q., Kang R., Klionsky D.J., Tang D., Liu J., Chen X. (2023). Copper metabolism in cell death and autophagy. Autophagy.

[36] Jiang Y., Huo Z., Qi X., Zuo T., Wu Z. (2022). Copper-induced tumor cell death mechanisms and antitumor theragnostic applications of copper complexes. Nanomedicine.

[37] Lee D., Son H.G., Jung Y., Lee S.V. (2017). The role of dietary carbohydrates in organismal aging. Cell. Mol. Life Sci. CMLS.

[38] Iglesias J., Morales L., Barreto G.E. (2017). Metabolic and Inflammatory Adaptation of Reactive Astrocytes: Role of PPARs. Mol. Neurobiol..

[39] Morant-Ferrando B., Jimenez-Blasco D., Alonso-Batan P., Agulla J., Lapresa R., Garcia-Rodriguez D., Yunta-Sanchez S., Lopez-Fabuel I., Fernandez E., Carmeliet P. (2023). Fatty acid oxidation organizes mitochondrial supercomplexes to sustain astrocytic ROS and cognition. Nat. Metab..

[40] Comerota M.M., Gedam M., Xiong W., Jin F., Deng L., Wang M.C., Wang J., Zheng H. (2023). Oleoylethanolamide facilitates PPARα and TFEB signaling and attenuates Aβ pathology in a mouse model of Alzheimer’s disease. Mol. Neurodegener..

[41] Wójtowicz S., Strosznajder A.K., Jeżyna M., Strosznajder J.B. (2020). The Novel Role of PPAR Alpha in the Brain: Promising Target in Therapy of Alzheimer’s Disease and Other Neurodegenerative Disorders. Neurochem. Res..

[42] Kummer M.P., Schwarzenberger R., Sayah-Jeanne S., Dubernet M., Walczak R., Hum D.W., Schwartz S., Axt D., Heneka M.T. (2015). Pan-PPAR modulation effectively protects APP/PS1 mice from amyloid deposition and cognitive deficits. Mol. Neurobiol..

[43] Corbett G.T., Gonzalez F.J., Pahan K. (2015). Activation of peroxisome proliferator-activated receptor α stimulates ADAM10-mediated proteolysis of APP. Proc. Natl. Acad. Sci. USA.

[44] Chandra S., Roy A., Jana M., Pahan K. (2019). Cinnamic acid activates PPARα to stimulate Lysosomal biogenesis and lower Amyloid plaque pathology in an Alzheimer’s disease mouse model. Neurobiol. Dis..

[45] Roy A., Kundu M., Jana M., Mishra R.K., Yung Y., Luan C.H., Gonzalez F.J., Pahan K. (2016). Identification and characterization of PPARα ligands in the hippocampus. Nat. Chem. Biol..

[46] Raha S., Ghosh A., Dutta D., Patel D.R., Pahan K. (2021). Activation of PPARα enhances astroglial uptake and degradation of β-amyloid. Sci. Signal.

[47] Jia W., Peng Q., Su L., Yu X., Ma C.W., Liang M., Yin X., Zou Y., Huang Z. (2018). Novel Bioactive Peptides from *Meretrix meretrix* Protect *Caenorhabditis elegans* against Free Radical-Induced Oxidative Stress through the Stress Response Factor DAF-16/FOXO. Mar. Drugs.

[48] Yu X., Li H., Lin D., Guo W., Xu Z., Wang L., Guan S. (2021). Ginsenoside Prolongs the Lifespan of *C. elegans* via Lipid Metabolism and Activating the Stress Response Signaling Pathway. Int. J. Mol. Sci..

[49] Xiao X., Zhang X., Zhang C., Li J., Zhao Y., Zhu Y., Zhang J., Zhou X. (2019). Toxicity and multigenerational effects of bisphenol S exposure to *Caenorhabditis elegans* on developmental, biochemical, reproductive and oxidative stress. Toxicol. Res..

[50] Schulz T.J., Zarse K., Voigt A., Urban N., Birringer M., Ristow M. (2007). Glucose restriction extends *Caenorhabditis elegans* life span by inducing mitochondrial respiration and increasing oxidative stress. Cell Metab..

